# *EyeT4Empathy*: Dataset of foraging for visual information, gaze typing and empathy assessment

**DOI:** 10.1038/s41597-022-01862-w

**Published:** 2022-12-03

**Authors:** Pedro Lencastre, Samip Bhurtel, Anis Yazidi, Gustavo B. M. e Mello, Sergiy Denysov, Pedro G. Lind

**Affiliations:** 1 Dep. Computer Science, OsloMet - Oslo Metropolitan University, P.O. Box 4 St. Olavs plass, N-0130 Oslo, Norway; 2 OsloMet Artificial Intelligence lab, OsloMet, Pilestredet 52, N-0166 Oslo, Norway; 3 NordSTAR - Nordic Center for Sustainable and Trustworthy AI Research, Pilestredet 52, N-0166 Oslo, Norway

**Keywords:** Neuroscience, Saccades, Statistics

## Abstract

We present a dataset of eye-movement recordings collected from 60 participants, along with their empathy levels, towards people with movement impairments. During each round of gaze recording, participants were divided into two groups, each one completing one task. One group performed a task of free exploration of structureless images, and a second group performed a task consisting of gaze typing, i.e. writing sentences using eye-gaze movements on a card board. The eye-tracking data recorded from both tasks is stored in two datasets, which, besides gaze position, also include pupil diameter measurements. The empathy levels of participants towards non-verbal movement-impaired people were assessed twice through a questionnaire, before and after each task. The questionnaire is composed of forty questions, extending a established questionnaire of cognitive and affective empathy. Finally, our dataset presents an opportunity for analysing and evaluating, among other, the statistical features of eye-gaze trajectories in free-viewing as well as how empathy is reflected in eye features.

## Abstract (summary)

**Table:** 

Measurement(s)	Gaze position
Technology Type(s)	eye tracking device
Factor Type(s)	Luminosity • Screen distance
Sample Characteristic - Organism	Human eye
Sample Characteristic - Environment	laboratory facility
Sample Characteristic - Location	Municipality of Oslo

## Background & Summary

The popular saying “The eyes are the mirror of the soul” has some basis in scientific fact: our eyes are the only part of our sensory system whose dynamics during the information perception and processing can be directly seen. Because of that, eye movement data are used in diverse fields, such as marketing, to understand what catches people’s attention^1–3^; neurosciences, to assess the development stages of infants before talking ages^4^; artificial intelligence, to improve mechanisms of free-viewing^5^; virtual reality, to predict when people will load images^6^; adaptive interfaces, to improve the performance of partially automated vehicles^7^ or helping non-verbal movement-impaired people (e.g. people suffering from tetraplegia, brain paralysis and locked-in syndrome) to interact with computers^8,9^. In medicine, namely in neurology and psychiatry, electroencephalogram data can be useful to know ‘where’ some pathology may originate, but it is often not enough to quantify ‘how’ serious the pathology is. Different eye-tracking methods have been used in recent years to assess the severity of such pathologies as dementia^10^, Parkinson’s and Huntington’ disease^11,12^, simple migranes^13^, or the effect of ketamine and other drugs on the nervous system^14^.

In general terms, the diagnosis protocol based on gaze tracking technologies^15^ has the aim of finding statistical features of the two main components of gaze dynamics, that are fixations and saccades. Fixations concern the periods when the eyes focus on a small region of the image. Typically, parts of eye-gaze trajectories corresponding to fixations are characterized by small fluctuations around the focus point^16^. As for saccades, they alternate with fixation events and are characterized by fast gaze point relocations. This classification is important for several applications, for example, for systems that allow people with movement impairments to interact with computers^17,18^.

Another important feature of human eyes is the pupil size. Its changes are correlated with the intensity of information processing and our cognitive states in general^19^. Pupil dilation indicates arousal^20^, emotional state (fear, sympathy or hostility), and sudden dilation may indicate stress or anxiety. For these reasons, pupil size measurements are used in psychology, psychiatry, and psycho-physiological research^21^.

**Fig. 1:**
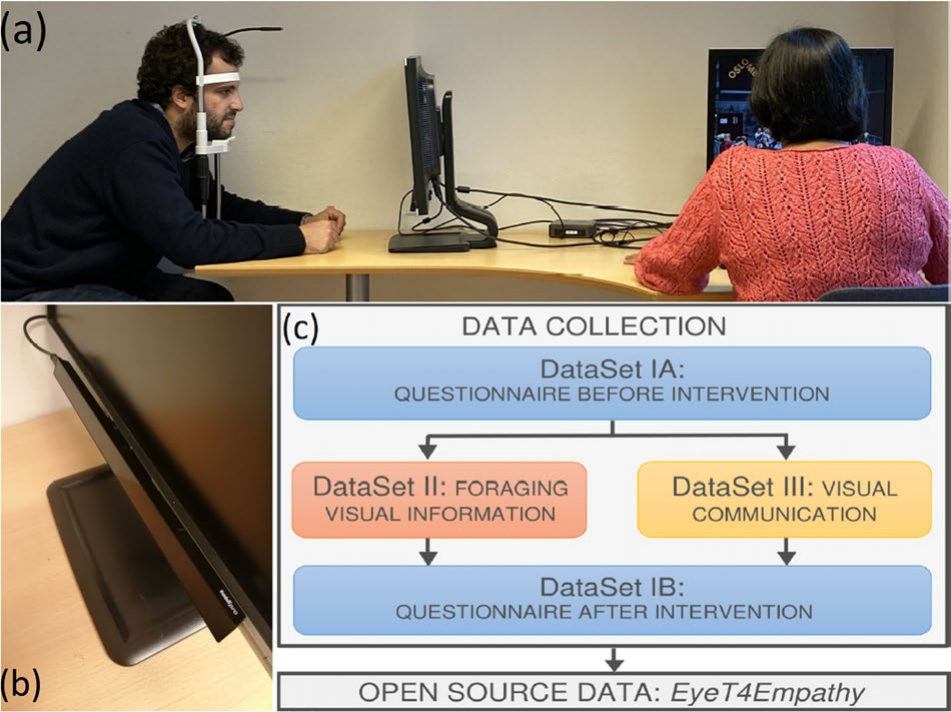
Summary of the data collection process to build the two *EyeT4Empathy* datasets: (**a**) Experimental setup of the eye-tracker; (**b**) Close-up of the eye-tracker equipment; (**c**) Steps of the data collection process.

Of particular interest is the use of pupil dilation as a marker of empathy. Typically, our pupil dilates when we perceive empathy towards someone, e.g. when we perceive sadness in someone’s face^22^, when we share a laugh with someone^23^, or when we hear someone crying^24^. A recent study on empathy was performed at the Oslo Metropolitan University, in which healthy adults are put in a circumstance where they are forced to communicate through a computer using only eye movements^25^. Figure 1a,b shows the experiment set-up and a close-up of the eye-tracker used, respectively.

The experimental methodology comprehended a test group, in which the participants were asked to use eye movements on a letter cardboard to write sentences, and a control group, in which the participants were asked to identify objects and/or shapes in an image constructed with random pixels. This latter activity we term “foraging for visual information”. To assess the level of empathy towards non-verbal movement-impaired people, all participants in both groups answered a questionnaire before and after the intervention. The data collected with the questionnaires were stored as “Data Set IA” (before intervention) and “Data Set IB” (after intervention). The data collected with the control group, doing foraging of visual information, were stored as “Data Set II”, whereas the data collected from the test group, doing gaze typing, were stored as “Data Set III”. The three data sets composed the dataset we call *EyeT4Empathy*. See Fig. 1c and Table 1.

**Table 1:** Statistics of the datasets in the *EyeT4Empathy* dataset.

	Total	DataSet IA	DataSet IB	DataSet II	DataSet III
Number of participants	60	60	60	30	30
Number of time-series	502	n.a.	n.a.	142	360
#TotPoints	4.8 × 10^6^	60	60	1.1 × 10^6^	3.8 × 10^6^
#DataPoints	4.2 × 10^6^	60	60	9.2 × 10^5^	3.3 × 10^6^
#FixationPoints	2.5 × 10^6^	n.a.	n.a.	6.0 × 10^5^	1.9 × 10^6^
#SaccadePoints	9.2 × 10^5^	n.a.	n.a.	1.7 × 10^5^	7.6 × 10^5^
#UnclassifiedPoints	7.9 × 10^5^	n.a.	n.a.	1.5 × 10^5^	6.4 × 10^5^

Here, ‘#TotPoints’ is the total number of points composing each time-series, cumulated over all 502 time-series. ‘#DataPoints’ is calculated from ‘#TotPoints’ by subtracting the missing values of the gaze position. The numerical values for which ‘#DataPoints’ accounts for can fit into 3 categories, based on the type of gaze behaviour: ‘Fixation’ (short movements around a fixed area), ‘Saccade’ (faster and longer movements between two fixated areas) or ‘Unclassified’ if they don’t fit any of the other two criteria. Thus ‘#DataPoints = #FixationPoints + #SaccadePoints + #UnclassifiedPoints’.

Our dataset differs from other open-access datasets^26–28^, in both the test and control group. The data corresponding to the test group can be used e.g. to assess how people can communicate with their eyes, while the control group can be used to see the “pure process” of foraging for visual information without being disturbed by finding objects. This last group allows to gather data of eye-movements while the participant is engaged only with trying to find patterns, enabling the study of the dynamical properties of gaze trajectories in a foraging mode, i.e. the eye-gaze is forced to move through the image in a similar way as a roaming predator without finding its prey.

The study of the mechanism humans employ to forage for visual information is crucial to answer fundamental questions of eye-movements. For over 20 years to the publishing of this study, it has been published repeatedly that gaze follows a Lévy-flight like dynamics^29–32^. This is similar to typical foraging dynamics, from albatroses or sharks foraging for food^33,34^ to the strategies of hunter-gatherers^35^ or the way children explore a theme park^36^. This is in direct contradiction to the cannon distinction between fixations and saccades. Such distinction has the underlying assumption that gaze-trajectories are modelled by an intermittent process (also called “composite random walk”)^37^, which is composed by two alternating different processes, with a feed-flight structure (see ref. ^38^ for details).

This question has implications beyond simple mathematical curiosity. The distinction between saccades and fixations is crucial in the field, with several algorithms proposed to distinguish between them. The properties of saccades and fixations are also used in distinguishing individuals with autism^10^, ADHD^39^, among others. On the other hand, the Hurst exponent^40^ has been proposed as a defining characteristic of gaze trajectories. This quantity is well defined only for Lévy flight type of dynamics and not for an intermittent process. Nonetheless, it has been suggested to be robust fundamental quantity of eye-movements^41,42^ and an efficient way to classify individuals^43^. Compared to other datasets, the one presented here extends the time participants are engaged in searching for patterns (typically 60 seconds). This is particularly relevant since gaze trajectories have been shown to have a dynamic that changes with viewing time (saccades are longer and more frequent when participants first encounter an image)^44^.

With this dataset, several studies can be done. One can, for example, use AI-methods to replicate gaze trajectories^45^ in order to generate large anonymous data-sets or study potential limitations of AI-methods; use natural language processing to improve autocorrect functions for gaze typing; study algorithms of classification of gaze movements as “saccades” or “fixations”, or use pupil diameter data along with the empathy questionnaires to study this already proposed correlation^22^.

While similar experiments of our control group were done previously^27,28^, they were done by much shorter periods of time. The inclusion of empathy levels allows us to assess an important psychological dimension. In what gaze typing is concerned, previous studies have described an optimized algorithm for gaze typing focused on improving its speed^46^. Still, some questions remain on how to optimize the task based on the presence of spacial noise.

Eye-tracker has also been increasingly used for emotion recognition^47^. Through the empathy questionnaire, this dataset can provide novel insights into to the already proven dependency from gaze dynamics and empathy^48^. It can also relate it with the attention of each participant by evaluating the respective accuracy or their writing. Besides the focus on disorders such as ADHD^39^, the increase in online learning and remote working has created the need to evaluate patterns consistent with close attention to a computer task.

The rest of the paper is organized as follows. First, in Section “Methods”, we will describe the two groups of participants and their prescribed tasks, the experimental set-up, design and protocol, hardware and the algorithm of saccade/fixation classification and the ethical considerations. In Section “Data records” we describe how the data was recorded and is organized. Considerations about hardware, equipment and experimental conditions are presented in Section “Technical validation”. Finally, Sections “Usage notes” and “Code availability” respectively contain the main instructions to assist other researchers to use the data sets in their research activities and the code to properly process the data and rebuild the results and figures presented in this paper.

## Methods

### Experimental design and protocol

The experiment was conducted in the AI Lab of Oslo Metropolitan University (Norway).

### Participants in the control and test groups

We performed both interventions, gaze typing and foraging for visual information, with 60 participants from 20–40 years of age. Most of the participants were students. Out of 60 participants, 4 were nurses by profession and a part time student and the remaining 56 were bachelors and masters students from University of Oslo, NTNU and Oslo Metropolitan University. The data was collected at Oslo Metropolitan University. Each session was recorded separately, and approximately 10 different data sets of eye-gaze trajectories were collected for each participant resulting in a total of 502 data-sets. Each participant answered twice the questionnaire for assessing the empathy level, before and after the respective intervention. Eye-tracker data with less than 600 data points or with over 60% NaN were not included in the total 502 data-sets.

### The task for the control group: free viewing

**Fig. 2:**
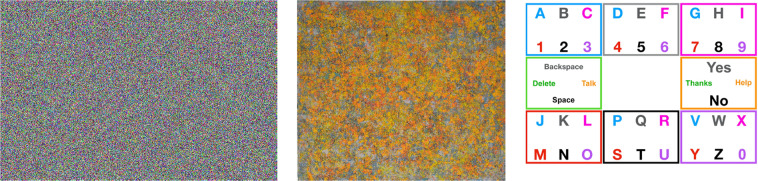
Stimuli shown to the participants. The left image and the middle one are shown to participants performing the “foraging for visual information” task, while the right most stimulus is shown to participants performing the “gaze typing” task.

**Fig. 3:**
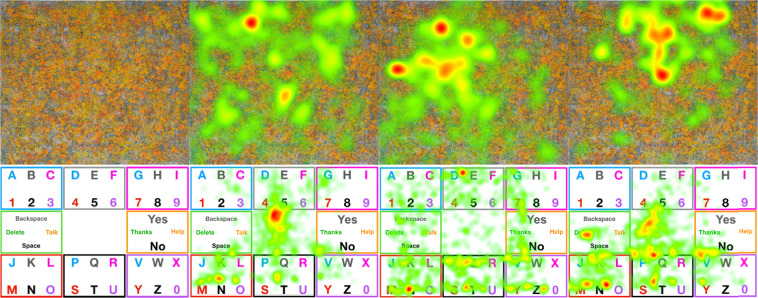
(Top): Image to assess foraging of visual information (reproduced with permission from the author) and three illustrative examples of heatmaps representing eye-gaze trajectories in three interventions. (Bottom): Image to assess gaze typing through gaze typing, using a letter board and three illustrative examples of heatmaps representing eye-gaze trajectories. Here, the density of fixation events land several times between letters to maximize information gain from multiple items.

In the control group, participants were asked to identify shapes in two images (stimuli). See Fig. 2 (left and middle). Each stimulus was shown twice, during one-minute intervals, alternating between trials. Illustrative output of the eye-gaze trajectories collected during these trials is shown in Fig. 3 (top). After the recording, each participant was asked about what they saw in the image. In the process of recording, we encouraged the participants to take a short break between trials (approx. 10–30 seconds).

### The task for the test group: gaze typing

For the test group, we started by explaining to each participant the uses of eye-tracking and how non-verbal movement-impaired people rely on this device to communicate and run their daily lives. This group was trained to use the digital E-tran board and the eye-tracker to write messages on the computer screen. See Fig. 2 (right). After this training, the participants were requested to write sentences, using the eye-tracking interface and the E-tran board exclusively. The sentences were the following ones: “Type your name”, “Can you help me?”, “Can you take me to the restroom?”, “I am hungry, can you do me some eggs?”, “I am uncomfortable. Can you change my position?”, “I am in pain. Can you give me medication?”. Results of the trajectories on the E-tran board are shown in Fig. 3 (bottom).

### Tasks to assess empathy: an extended questionnaire from QCAE

We have used and expanded a thirty-one questions questionnaire called questionnaire of cognitive and affective empathy (QCAE)^49^. The extended questionnaire has nine additional questions, focusing on the empathy towards people with motion disabilities^25^. The results with the extended questionnaire showed a more significant difference between control and test groups^25^. Our dataset includes the empathy scores and the answer to all individual questions in the extended questionnaire and the standard QCAE. This is true for both data sets IA and IB.

### Hardware and software

The hardware consisted of an eye-tracker *Tobii Pro X3–120* and the software *Tobii Pro Lab Presenter Edition* (Fig. 1b). The eye-tracker has an infrared light projector and a camera. The projector releases a pattern of light that is reflected by the eye gaze. The camera component of the device captures the coordinates of eye movements and helps collect the eye-gaze data of the users. This particular eye-tracker is a good compromise between the typical equipment used in the context of universal design and those one would like to use for studying the kinetic properties of eye-movements. While the first are typically inexpensive and as small as possible, with frequencies around 40 or 60 Hz, the latter prioritize accuracy and a high sample frequency at the expense of both affordability and portability.

Before recording the data, the person responsible for data collection provided the explanation of the experiment to the participants. We also explained the participants how to maintain their posture during the experiment, to ensure the quality and accuracy of the data. To stabilize the head movement, we had chin and forehead rest tools (Fig. 1a) from the eyes to the device within a distance between 47 and 70 cm, with a mode around 60 cm. See Table 2. The room chosen for the procedure received no sunlight and the luminous flux per unit area, also called luminance, was kept constant at 115 lx. These light conditions were considerable lower than an overcast which has around 1000 lx.

**Table 2:** Distance to the screen in millimeters (“Dist.”) and number of trials (“#Trials”) per participant (“ID”).

ID	Dist.(mm)	#Trials	ID	Dist.(mm)	#Trials	ID	Dist.(mm)	#Trials	ID	Dist.(mm)	#Trials
1	743	8	16	509	4	31	811	8	46	743	4
2	609	5	17	557	8	32	746	3	47	536	8
3	743	16	18	568	4	33	535	9	48	651	4
4	609	9	19	674	8	34	568	4	49	550	8
5	743	24	20	620	4	35	587	8	50	751	4
6	609	13	21	507	8	36	685	4	51	570	8
7	743	32	22	521	4	37	510	8	52	526	4
8	609	17	23	583	8	38	726	4	53	553	8
9	743	40	24	734	4	39	579	8	54	524	4
10	628	4	25	463	8	40	626	4	55	572	8
11	743	49	26	475	4	41	586	8	56	515	4
12	544	4	27	783	8	42	622	4	57	550	8
13	546	8	28	586	4	43	486	8	58	575	4
14	576	4	29	654	8	44	523	4	59	533	8
15	552	8	30	716	4	45	539	8	60	546	4

Distance to the screen is calculated automatically by the eye-tracker.

The native algorithm of classification of saccades and fixations is described in detail by the manufacturer (see ref. ^50^) and generally follows the approach introduced in ref. ^51^. In a first iteration, the algorithm classifies data points into fixations and saccades based on a velocity threshold of 30°/s (degrees of visual angle per second). Afterwards, it merges fixations that are close: a set of points in between two fixations is re-labeled as fixations if they they are separated in space and time by no more than 0.5° and 75 *ms* respectively. Finally, a minimum fixation time is introduced (60 ms) and fixations shorter than it are discarded. Data points that do not have a saccadic velocity nor form a fixation during 60 ms are labelled ‘Unclassified’.

### Ethical considerations

For the ethical consideration of data, an authorization was provided by *Norsk senter for forskningsdata* (NSD), which allows for the collection and publication of data (application number 119986). In compliance with ethical requirements, participants’ personal information (full name and contact information) was deleted after the procedure.

## Data Records

The dataset is available in the FigShare and distributed under a Creative Commons license. The dataset of eye-tracking movements^52^ is organized in 502 files, each one with a name following the syntax:


EyeT_group_AAA_image_name_BBB_participant_CCC_trial_DDD.csv.


Here, AAA can take the values dataset_II or dataset_III, depending if the participant performed the “foraging for visual information” or the “gaze typing” experiment, respectively. As for BBB, it can take the value of letter_card, grey_blue or grey_orange. The different stimuli are show in Fig. 3. CCC stands for the participant ID number in a given test/control. The number is sequential and enables to find the files corresponding to the same person, without identifying the participant.

Finally, DDD is the trial number for each participant, typically around 4 for the control group to around 12 for the test group. The exact number of trials for each participant depended on their availability. Each eye-tracker data record is composed by several columns, an explanation of which can be found in the same folder, in the files named columns_explained.pdf and coordinate_system.pdf.

The eye-tracker raw-data^53^ as extracted directly from the eye-tracker can also be found with the syntax:

ParticipantCCC.tsvwhere, as before, CCC represents the participant number. Participants with an even ID-number performed the “foraging for visual information”, while participants with an odd ID-number performed “gaze typing” experiment.

The empathy assessment questionnaire contains the answers to each individual question and the final empathy score^54^. The file name of each questionnaire is just:

Questionnaire_AAA.csvwhere AAA can take the values datasetIA or datasetIB depending on whether it is a questionnaire before or after the eye-tracker experiments. The list with all the questions can be found in the same directory.

## Technical Validation

One of the most fundamental aspects of data quality is the reliability of the eye-tracker used. Tobii Pro X3–120 Eye Tracker is used in state-of-the-art research all over the world in the fields of medicine and cognition^55,56^, robotics and informatics^57–59^, marketing^60^ or educational sciences^61^. It is also noticeable that the Tobii Pro X3–120 eye-tracker is also among the best devices used in the field of Universal Design^62^, where it was used to develop universally designed solutions, i.e. suited to people with and without specific disabilities. This allows access to detailed statistics of the gaze-trajectories while ensuring that subjects in the test-group face the similar difficulties of non-verbal movement-impaired individuals interacting with a computer.

The experiment was performed with a head stand designed to keep the head position fixed during the experiment. Furthermore, luminosity conditions and distance to the measurement device were calibrated in order to minimize measurement errors. A five-point calibration and validation session, provided by Tobii’s software^63^, preceded every data collection. Moreover, according to the product specifications, in the luminosity conditions of the experiment, taking in consideration the distance to the screen and screen length, the mean error should be inferior to 1° of the visual angle. See above in section “Hardware and Software”.

**Fig. 4:**
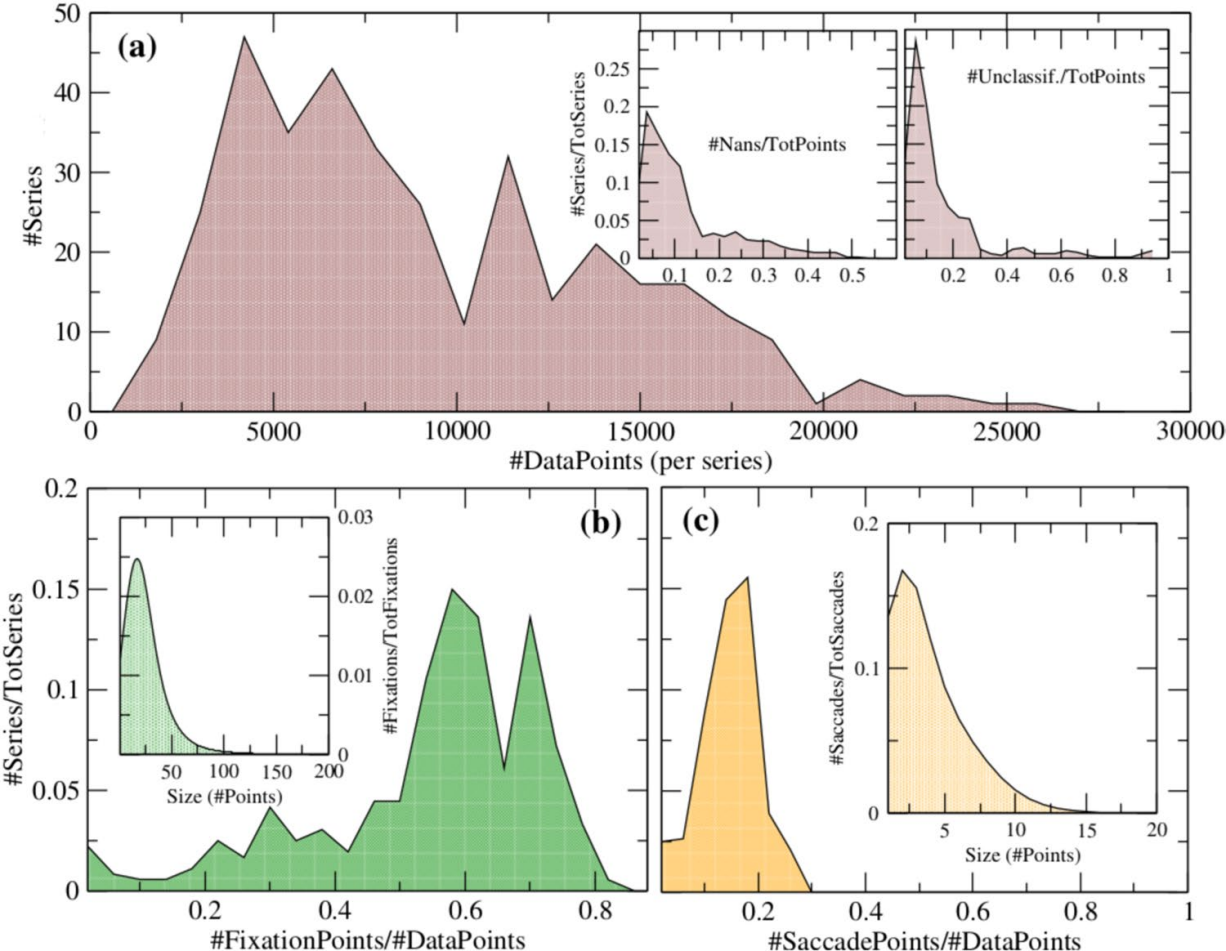
Overview of the data sets II and III together: (**a**) Histogram of the number of series (“#Series”) having a certain number of points (“#DataPoints”); the insets indicate the fractions of non-available numbers (“#Nans”) and the fraction of unclassified points (“#Unclass.”) from the total number of points (“TotPoints”). Here TotPoints = #DataPoints + #Nans, while #DataPoints = #SaccadePoints + #FixationsPoints + #UnclassifiedPoints, where #FixationsPoints and #SaccadesPoints are the *number of points* classified as fixations and saccades respectively. The figure also shows the histogram of the number of points from the total number of data points belonging to (**b**) fixations and (**c**) saccades, in one single series. The insets in (**b**) and (**c**) indicate the corresponding histogram of the size (number of points) of each single fixation and saccade, respectively. One individual fixation (resp. saccade) is defined as a set of consecutive points all labelled as “fixation” (resp. “saccade”). We call the total amount of fixations and saccades as “TotFixations” and “TotSaccades” respectively. Here “TotSeries” = 502, “TotPoints” = 4.8 × 10^6^, “FixationPoints” = 2.5 × 10^6^ and “SaccadePoints” = 9.2 × 10^5^. See also Table 1.

Missing samples records were labeled as ‘NaN’. This can happen due to blinks or due to loss of eye-image by the eye-tracker. The ratio of ‘NaN’ values in each time series is represented in Fig. 4a and it is observed that most time-series have less than 15% of the total points labeled as ‘NaN’. Pupil measurements were extracted at 40 Hz frequency and thus the number of NaNs in this part of the dataset is about triple than that of eye-movements. Nevertheless, pupil changes are typically much slower than that of eye-movements and thus it is considered that this frequency is adequate to study the time-evolution of pupil diameter^64,65^.

Another important aspect is the time-resolution of the eye-tracker. Capturing frames at a frequency of 120 HZ, the resolution is adequate to separate between saccades and fixations. We see in the inset of Fig. 4a that most series have less than 20% of data points ‘Unclassified’, i.e., not labelled as either ‘Saccade’ or ‘Fixation’.

For most time series fixations represent the majority of data points (Fig. 4b) while saccades represent between 10% to 20% of data points (Fig. 4c). The criteria used to distinguish from both saccades and fixations was provided directly from Tobii’s eye-tracking software. Fixations have on average 27 points each, while 85% of the saccades have typically only between two and ten consecutive points. See insets of Fig. 4b,c. These figures show that both regimes can be reasonably separated by the eye-tracker - the sampling rate is higher than the transition rate between fixations and saccades.

Notice that the size of the stimuli are different. See Table 3. To cope with the different sizes, we labelled as NANs all points of eye-gaze trajectories beyond the limits of the stimuli.

**Table 3:** Sizes of the stimuli and size of the screen during the experiment.

	Width (pixels)	Height (pixels)
Screen	1920	1080
Fig. 2 left	1662	1080
Fig. 2 middle	1245	1080
Fig. 2 right	1493	1080

## Usage Notes

The data processing of the data sets in the repository consisted mainly in the decomposition of each data sets into saccades and fixations, as described above. This decomposition was done using the software *Tobii Pro Lab Presenter Edition*. No other processing requirements were needed.

## Data Availability

The .csv files composing the dataset were extracted directly from Tobii Pro X3–120 Eye Tracker, as well as images such as heatmaps. The original raw-data files and the python code used to generate the .csv files, figures and statistics here are available at Figshare^66^.
